# Reduced Anxiety and Depression-Like Behaviours in the Circadian Period Mutant Mouse Afterhours

**DOI:** 10.1371/journal.pone.0038263

**Published:** 2012-06-15

**Authors:** Robert Keers, Inti Pedroso, Gerome Breen, Kathy J. Aitchison, Patrick M. Nolan, Sven Cichon, Markus M. Nöthen, Marcella Rietschel, Leonard C. Schalkwyk, Cathy Fernandes

**Affiliations:** 1 MRC SGDP Centre, Institute of Psychiatry, King’s College London, London, United Kingdom; 2 MRC Mammalian Genetics Unit, Harwell, Didcot, Oxfordshire, United Kingdom; 3 Institute of Neuroscience and Medicine (INM-1), Structural and Functional Organization of the Brain, Genomic Imaging, Research Center Juelich, Juelich, Germany; 4 Department of Genomics, Life & Brain Center, University of Bonn, Bonn, Germany; 5 Institute of Human Genetics, University of Bonn, Bonn, Germany; 6 Department of Genetic Epidemiology in Psychiatry, Central Institute of Mental Health, University of Mannheim, Mannheim, Germany; Vanderbilt University, United States of America

## Abstract

**Background:**

Disruption of the circadian rhythm is a key feature of bipolar disorder. Variation in genes encoding components of the molecular circadian clock has been associated with increased risk of the disorder in clinical populations. Similarly in animal models, disruption of the circadian clock can result in altered mood and anxiety which resemble features of human mania; including hyperactivity, reduced anxiety and reduced depression-like behaviour. One such mutant, after hours (*Afh*), an ENU-derived mutant with a mutation in a recently identified circadian clock gene *Fbxl3,* results in a disturbed (long) circadian rhythm of approximately 27 hours.

**Methodology:**

Anxiety, exploratory and depression-like behaviours were evaluated in *Afh* mice using the open-field, elevated plus maze, light-dark box, holeboard and forced swim test. To further validate findings for human mania, polymorphisms in the human homologue of *FBXL3,* genotyped by three genome wide case control studies, were tested for association with bipolar disorder.

**Principal Findings:**

*Afh* mice showed reduced anxiety- and depression-like behaviour in all of the behavioural tests employed, and some evidence of increased locomotor activity in some tests. An analysis of three separate human data sets revealed a gene wide association between variation in *FBXL3* and bipolar disorder (*P* = 0.009).

**Conclusions:**

Our results are consistent with previous studies of mutants with extended circadian periods and suggest that disruption of *FBXL3* is associated with mania-like behaviours in both mice and humans.

## Introduction

Disturbances to the circadian rhythm have long been hypothesised to play a pivotal role in the pathophysiology of bipolar disorder. Abnormalities in sleep/wake cycle, appetite and social rhythms are key features of both manic and depressive episodes and core symptoms, including disturbances of mood, show considerable diurnal variation [1]. Moreover, there is some evidence to suggest that there are significant differences between the chronotypes of bipolar disorder patients and healthy controls [2]–[4].While circadian rhythm disruption may precipitate episodes of mania, normalisation of circadian rhythms may be critical for mood stabilisation [5]. Indeed several studies have suggested that the therapeutic benefits of treatments for bipolar disorder including antidepressants and mood stabilisers may depend on their effects on the circadian rhythm [6]–[14].

In mammals the circadian rhythm is endogenously generated in the suprachiasmatic nucleus (SCN) of the hypothalamus and consists of complex feedback loops of transcriptional activation and repression. Studies of clinical populations suggest that variation in genes implicated in the circadian clock including *CLOCK*, *PER3*, *BMAL1*, *TIMELESS*, *NPAS2*, *NR1D1, RORB* and *GSK3β* may contribute to the genetic component of bipolar disorder [15]–[20]. While the exact mechanism by which these polymorphisms affect susceptibility to bipolar disorder remains unknown, animal studies suggest that disruption to the circadian clock may be associated specifically with the manic phase of the disorder.

Mania-like behaviour, including hyperactivity, reduced anxiety or increased risk taking behaviour, and reduced depression or euphoria have been reported in some animal models of circadian dysfunction [21]–[24]. These similarities with human mania phenotypes are further supported by data suggesting that treatment with the mood stabiliser lithium can ameliorate both human and mouse behaviours [25]. Lithium itself can modulate the circadian clock by inhibiting GSK3β, a key regulator of circadian functioning. However, mania-like behaviour is not the only consequence of altered circadian clock function as some circadian clock mutants do not show mania-like behaviours [1], [25] and in mice harbouring an ENU-induced mutation in *Clock*, additional behaviours such hyperhedonia, altered sleep patterns and increased preference towards cocaine have been reported [26].

After hours (C3H.Cg-Fbxl3<Afh>/H; *Afh*) is an ENU-derived mutant with a lengthened circadian rhythm of wheel-running activity of approximately 27 hours, similar to the *Clock* mutant [27]–[28]. Although the long circadian period of *Afh* was originally detected under un-entrained conditions, under entrained conditions *Afh* mice do present with the same phase delay analogous to the chronotype of ‘eveningness’ in humans [28].

The causative mutation has been identified as a Cys^358^Ser substitution in *Fbxl3*, an F-box protein with leucine-rich repeats which participates in the ubiquitin dependent degradation of CRY, a key transcriptional repressor within the circadian molecular oscillator [29].

The aim of the current report was to investigate the effects of this mutation on anxiety and depression-like behaviour by comparing the behaviour of male *Afh/Afh* and *Afh/+* mice and their wildtype (+/+) littermates using a battery of validated tests. Consistent with previous findings in *Clock* mutant mice, *Afh* mutants showed reduced anxiety and depression-like behaviour. In order to translate findings from *Afh* mutants to human mania we investigated the association between variation in the human orthologue of *Fbxl3* in three large genome-wide association studies [30]–[32] of bipolar disorder and found evidence for association.

## Methods

All housing and experimental procedures were performed in compliance with the UK Home Office Animals Scientific Procedures Act 1986 (Home Office project licence number 70/7184).

### Animals

*Afh* mice were created at the Medical Research Council (MRC) Mammalian Genetics Unit (Harwell, UK) using ENU mutagenized BALB/c males which were crossed to C3H/HeH females and subsequently backcrossed for more than 10 generations to C57BL/6J [28]. 15 male mice homozygous for the mutation (*Afh/Afh*), 9 male mice heterozygous for the mutation (*Afh/+*) and 13 male wildtype (+/+) mice [28] were bred at the MRC Mammalian Genetics Unit (Harwell, UK) and shipped to the Institute of Psychiatry, King’s College London for behavioural testing. The mice were individually housed in standard cages (40×25×12 cm) with food (Rat & Mouse No 3 diet, Special Diet Services, Essex, UK) and water available *ad libitum*. Mice were singly housed during behavioural testing to minimise confounding effects of group housing and establishment of social hierarchies [33]. The housing room was maintained at constant room temperature (21°C) and humidity (45%) and kept under a regular light/dark schedule with lights on from 08∶00 to 20∶00 hours. Sawdust (Litaspen premium) and nesting materials (Sizzlenest, Datsand, Manchester, U.K.) in each cage were changed once every two weeks but never on the day before, or the day of, testing to minimize the disruptive effect of cage cleaning on behaviour. The mice were between 26 and 34 weeks old at the start of testing (mean age 29.7, SD 2.9 and mean weight 30.5, SD 2.6). All behavioural tests were performed in the light phase between 09∶00 and 17∶00 hours in a counterbalanced order across the genotype groups. We have previously shown that the behaviours being measured in this study are not altered by either testing time of day or single housing C57BL/6 J, the wildtype background strain [34]. Experimenters were blind to the genotypes of the animals both during testing and subsequent scoring of the recorded behaviour. In order to confirm genotypes of the mice tested, mice were re-genotyped as described previously [28] behavioural testing.

### Measures of Anxiety-like Behaviour

The open field test [35], light dark box [36] and elevated plus maze [37] were used to measure anxiety-like behaviours in the mice. Each test measures the conflict between rodents’ exploratory behaviour and aversion to open and brightly lit areas. Decreasing entries and time spent in the anxiogenic areas is indicative of increased anxiety-like behaviour and is reversed by treatment with anxiolyics and antidepressants [38].

### Open Field

The open field apparatus consisted of a square white acrylic arena measuring 72 × 72 × 33, under artificial light (40 lux). Animals were allowed to explore the arena for five minutes while their movements were detected using an automated tracking system (Ethovision, Noldus Information Technologies, Wageningen, The Netherlands) connected to a video camera positioned overhead. Using Ethovision, the frequency of entries into, and the time spent in, the central zone of the arena (18×18 cm) were extracted. The total distance (cm) travelled and the mean velocity (cms per second) in the non-threatening, outer zone of the open field was also extracted to provide a measure of locomotor activity which is not confounded by any changes in anxiety. The number of faecal boli and instances of urination were recorded at the end of each trial and the arena wiped clean with 1% Trigene solution.

### Light Dark Box

The light dark box was made of white acrylic and consisted of an arena (45×21×21 cm) partitioned into two compartments: a light compartment (30×21×21 cm under 85 lux illumination) and a dark compartment (15×22×22 cm, under 15 lux illumination). A small entry within the compartment partition (5×7 cm) allowed each mouse to move between chambers freely. Animals were placed in the dark compartment and their movement tracked for 5 minutes using tracking software (Ethovision, Noldus Information Technologies, Wageningen, The Netherlands) connected to a video camera positioned overhead. The mean velocity (cm per second) and total distance (cm) moved in the dark compartment were extracted from the tracking software to provide a measure of locomotor activity not confounded by any changes in anxiety. As the tracking system is less sensitive at determining a transition between the compartments, particularly when a mouse sits between the compartment partition, the number of entries into, and the time spent in, the light compartment were manually scored. Entry to either compartment was defined as all four paws in one compartment. The number of faecal boli and instances of urination were recorded at the end of each trial and the arena wiped clean with 1% Trigene solution.

### Elevated Plus Maze

The elevated plus maze (EPM) was constructed from black (floor) and transparent (walls) acrylic and consisted of two closed (30×5 cm) and two open arms (30×5 cm) extending from a central platform (5×5 cm). One set of arms, opposing one another, were enclosed by a 15 cm wall of transparent acrylic (‘closed arms’), while the other set was open with a ledge of 0.5 cm either side of the arms (‘open arms’). The maze was elevated 40 cm from the ground on a transparent acrylic stand. Light intensity around the maze was set at approximately 20 lux. Animals were placed in the central platform, facing towards a closed arm, and allowed to explore the maze freely for 5 minutes while their movement was tracked using an automated tracking system (Ethovision, Noldus Information Technologies, Wageningen, The Netherlands) connected to a video camera positioned overhead. Entries to the closed and open arms were manually coded from videotape. Entry into an arm was scored when all four paws of the animal had entered an arm. Exit from an arm was scored when the forepaws of the mouse had left the arm. The number of entries to each arm and time spent in each arm were extracted for analysis. Distance (cm) moved and speed (cm per second) of each mouse in the closed arms was extracted from the tracking software to provide a measure of locomotor activity not confounded by any changes in anxiety. The number of faecal boli and instances of urination were recorded at the end of each trial and the arena wiped clean with 1% Trigene.

### Holeboard

The holeboard measures activity and exploration in a novel arena [39]. The Truscan Photo Beam Activity System (Coulbourn Instruments, Whitehall, PA) was used, which consists of an arena (25.4 cm square) and a nose poke floor with 16 holes (4×4 array) with sensor rings to track movement. The beams are spaced 1.52 cm apart providing a 0.76 cm spatial resolution. The ambient lighting in the test room was 20 lux. Animals were placed in the arena and the movement, the number of nose pokes and the time spent nose poking were recorded for 5 minutes using the Truscan program. The frequency of entries and time spent in the centre of the holeboard were also measured as well as activity measures of average speed and distance travelled. The number of faecal boli and instances of urination were recorded at the end of each trial and the arena wiped clean with 1% Trigene.

### Forced Swim Test

In the forced swim test mice were placed for 5 minutes in a transparent cylinder measuring 20 cm in diameter and filled with room temperature water to a depth of 15 cm. An increase in immobility over time is considered to be associated with behavioural despair and therefore increased depression-like behaviour which is ameliorated by treatment with antidepressants [40]. Consistent with previous studies of circadian clock mutant mice [22] two trials were conducted 24 hours apart. Activity in both trials was recorded using cameras positioned both overhead and perpendicular to the forced swim test and behaviours scored manually from videotape using Ethovision (Ethovision, Noldus Information Technologies, Wageningen, The Netherlands). Struggling (defined as a vertical posture with limb movement), swimming (defined as a horizontal posture with limb movement), paddling (defined as horizontal posture with minimal movement in one limb) and absolute immobility were scored. Mean duration and latency of immobility were extracted from the manual scores. (Faecal boli were removed at the end of each trial).

### Corticosterone Assay

Approximately 50 µl of whole blood was collected exactly 24 hours prior to forced swim test (baseline or pre-stress measure) between 10∶00 and 16∶00 hours and again 30 min after each of the first forced swim trials (post-stress measure). Forced swim testing was performed between 10∶00 and 16∶00 hours. Blood collection was completed within 120 s after removing each mouse from its cage. Blood obtained via tail sampling was collected into potassium- EDTA microvette CB 300 tubes (Sarstedt, Nümbrecht, Germany), stored on ice and centrifuged with 12,000 rpm at 4°C for 10 min to separate out the plasma. Blood plasma was stored at –20°C. Plasma corticosterone levels were determined in duplicate from 20 µl of plasma using commercially available enzyme immunoassay kits (Assay Designs, Ann Arbor, U.S.A.); sensitivity 30 pg/ml.

### Statistical Analyses

The effect of genotype on behavioural and corticosterone measures was analysed using ANOVAs and post-hoc t tests. A Box-Cox transformation was used to normalise the forced swim data as this was not normally distributed. All reported p values are two tailed. All statistical analyses were performed using STATA 10.0 [41].

### Analysis of Human Data Genome-wide Association

We used data from three independent genome-wide association studies (GWAS) of bipolar disorder [30]–[32]. Full details of each study have been published previously but briefly, 469,557 SNPs in 2,000 bipolar I and bipolar II cases and 3,000 combined controls were genotyped in the WTCCC study [30], 372,193 SNPs in 1461 bipolar I cases and 2008 controls were genotyped in the Sklar et al study [31] and 499,494 in 2411 bipolar I and bipolar II cases and 3613 controls were genotyped in the Chicon et al study [32]. We had formal access to the WTCCC data from EGA (http://www.ebi.ac.uk/ega/) and downloaded post-QC summary statistics of the Sklar et al ^30^ GWAS from http://pngu.mgh.harvard.edu/purcell/bpwgas/in December 2007. We extracted association p-values calculated under an additive model for all SNPs within 20 kb of the human orthologue of *Fbxl3* (Ensembl identifier: ENSG00000005812). For each GWAS the SNP p-values were combined to calculate a gene p-value using the software FORGE as previously described elsewhere [42] and that is freely available at http://github.com/inti/FORGE. We analyzed gene p-values by transforming the p-values into z-scores of a standard normal distribution and calculated a combined z-score by Z  =  Σz_i_/√N, where z_i_ are the studies’ z-scores and N the total number of studies.

## Results

### Open Field Test

**Figure 1:**
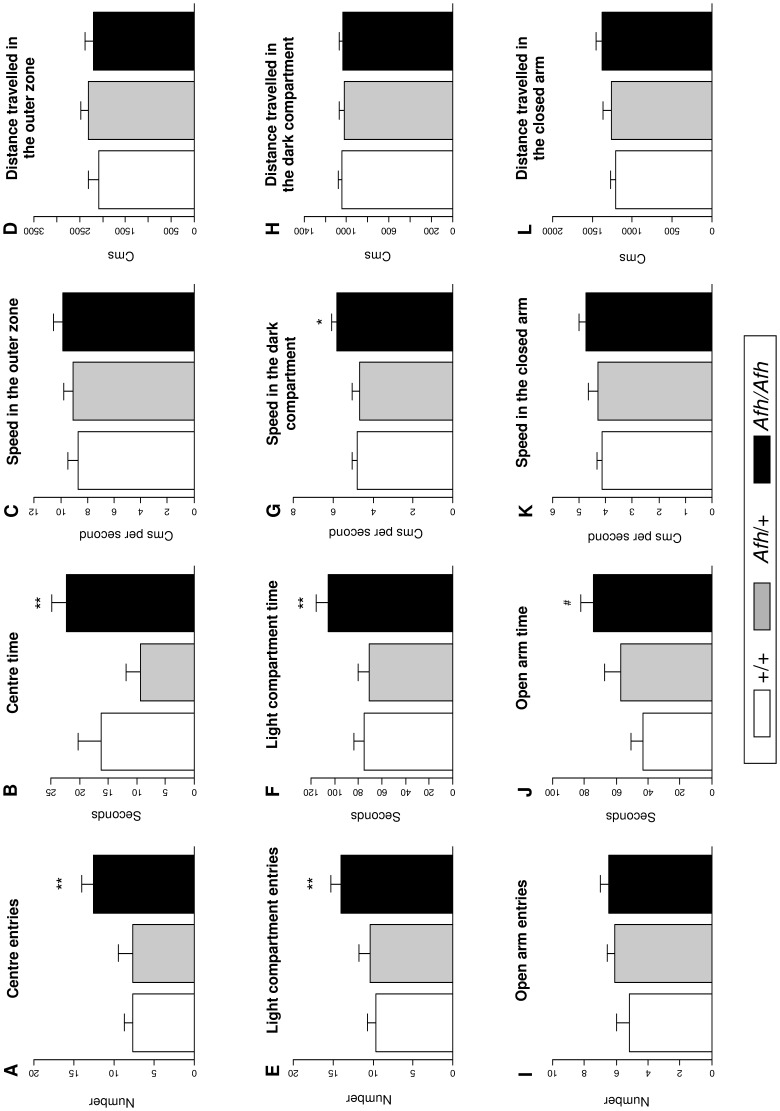
Anxiety-like behaviour is lower in after hours mice. In the open field test (A-D) *Afh*/*Afh* mice entered the central zone more frequently and spent more time there but did not differ in activity measures of speed and distance in the outer zone. In the light-dark box (E–H) *Afh*/*Afh* mice entered the light compartment more often and spent more time there and had increased speed in the dark compartment but did not differ in distance travelled in the dark compartment. In the elevated plus maze (I-L), *Afh*/*Afh* mice spent more time in the open arms but did not differ in their frequency of entry or closed arm activity measures. (Data shown is mean ± SEM). **P<0.01, *P<0.05, ^#^ *P* = 0.06; effects of genotype in posthoc t-tests (*Afh*/*Afh vs. Afh*/+ and +/+*)*.

There was a significant effect of genotype on both the number of entries (*F*(2,34)  = 4.46, *P*<0.01) and the time spent in the central area (*F*(2,34), *P* = 0.03). Specifically, *Afh/Afh* mice entered the centre more frequently and spent more time in this area than *Afh/+* and +/+ mice. Locomotor activity, measured as distance travelled and speed in the outer area of the open field, did not differ significantly between groups (Figure 1, a–d).

### Light Dark Box

There was a significant effect of genotype on the number of entries (*F*(2,34)  = 4.23, *P* = 0.02) and the time (*F*(2,34)  = 4.23 *P* = 0.02) spent in the light compartment of the light dark box (Figure 1). *Afh/Afh* mice entered the light compartment more frequently and spent more time there than either *Afh/+* or +/+ mice (Figure 1,e,f). Distance travelled in the dark area was unrelated to genotype (*F*(2,34)  = 0.10, *P*>0.1) however speed in the dark area was significantly higher in *Afh/Afh* than *Afh/+* and +/+ mice (*F*(2,34)  = 5.46, *P*<0.01) (Figure 1,g,h).

### Elevated Plus Maze

There was a significant effect of genotype on the time spent in the open arms of the elevated plus maze with +/+, *Afh/+,* and *Afh/Afh* mice spending progressively greater amounts of time in the open arms (*F*(2,34)  = 3.78 *P* = 0.03; Figure 1, i-l). Although there appeared to be an effect of genotype on the number of entries to the open arm this did not reach significance (*F*(2,34)  = 1.21, *P*>0.1). There was a trend to suggest that *Afh/Afh* mice were faster and travelled a greater distance when compared to +/+ and *+/Afh* mice in the elevated plus maze.

### Holeboard

**Figure 2:**
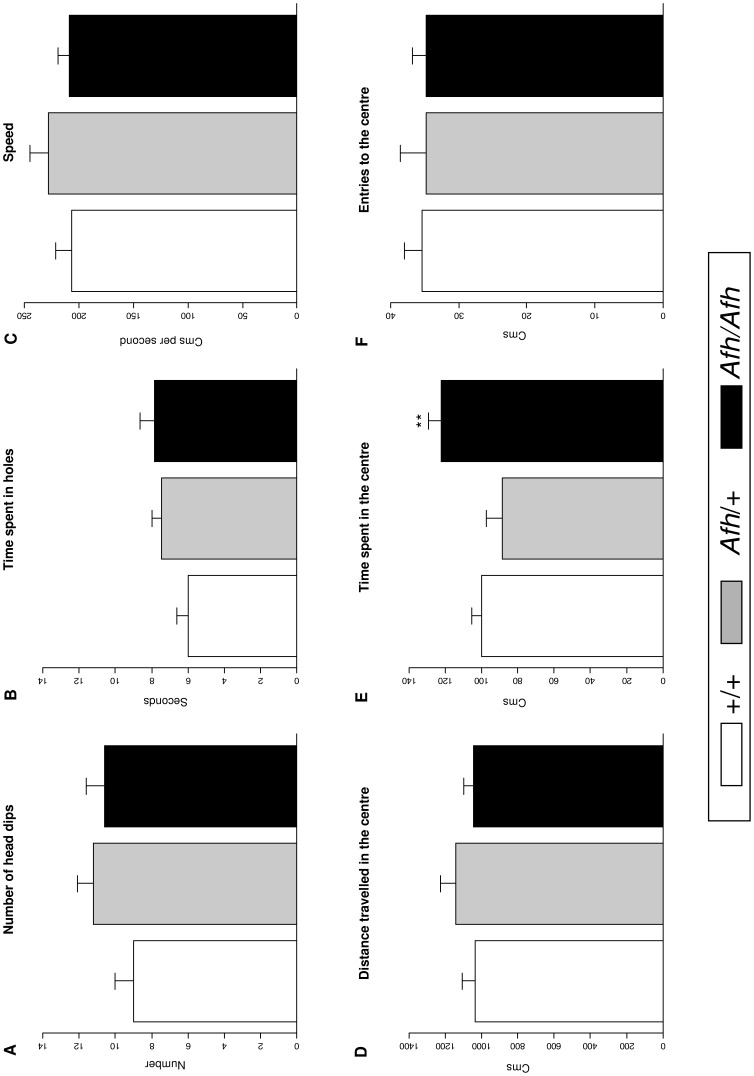
The effect of after hours genotype on exploratory and anxiety-like behaviour in the holeboard. Results from the holeboard test indicate no difference in exploratory behaviours (A–B) or overall activity (C–D). *Afh*/*Afh* mice spent more time in the centre (E) but did not differ in their number of entries (F). (Data shown is mean ± SEM). **, *P*<0.01 effects of genotype in posthoc t-tests (*Afh*/*Afh vs. Afh*/+ and +/+*)*.

Both the frequency of, and time spent, head dipping were unrelated to genotype, suggesting no effect of the mutation on explorative behaviour. Entries to the centre of the holeboard also did not differ by genotype. However, the time spent in the centre was significantly greater in *Afh/Afh* mice (*F*(2,33)  = 11.41, *P*<0.01; Figure 2).

### Forced Swim Test

**Figure 3:**
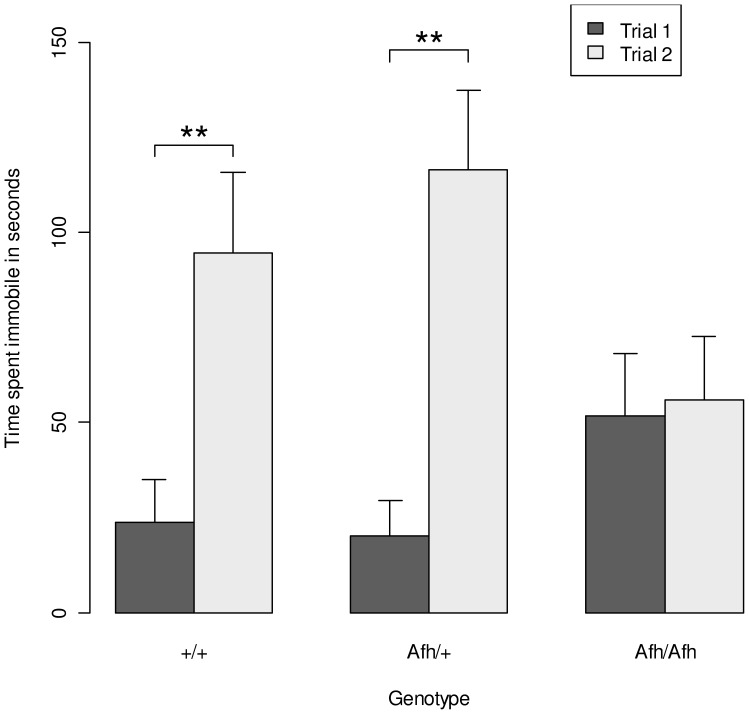
Immobility in the forced swim test is attenuated in after hours mice. Results from the forced swim test indicate no significant difference in immobility in trial 1 but the increase in immobility observed in trial 2 was not seen in *Afh/Afh* mice. (Data shown is mean ± SEM). **, *P*<0.01 results from posthoc t-tests comparing immobility in trial 1 *vs.* trial 2 in *Afh/Afh. Afh*/+ and +/+ mice.

In Trial 1 of the forced swim test 26 of the 37 animals tested showed immobility. The 11 mice that failed to show immobility did not differ significantly by genotype (χ^2^ = 0.42, *P* = 0.80). There were no effects of genotype on latency to immobility and although *Afh/Afh* appeared to show longer periods of immobility than both *Afh/+* and +/+ mice, this difference did not reach statistical significance (*t* = 1.80, *P* = 0.08, Figure 3).

Conversely in trial 2 of the FST, conducted 24 hours later, both *Afh/+* and +/+ mice remained immobile for a significantly longer period than those homozygous for the mutation (t = 2.2, *P* = 0.03). A repeated measures ANOVA indicated a significant genotype by trial interaction (*F*(2,68)  = 7.39, *P*<0.01). Specifically, while both *+/Afh* and +/+ mice were significantly more immobile in the second trial, there was no such change in the immobility time of *Afh/Afh* mice.

### Weight

There was a significant effect of genotype on weight prior to testing (*F* = 6.43, *P*<0.01) such that *Afh/Afh* mice were heavier than *Afh*/*+* and +/+ mice (*t* = 3.6, *P*<0.001). This effect was independent of age which did not differ significantly by genotype (*F* = 0.01 *P*>0.1). As weight may confound the relationship between genotype and behavioural measures, analyses were therefore repeated using ANCOVAs adjusting for weight prior to testing. All results remained significant.

### Corticosterone Response to the Forced Swim

**Figure 4:**
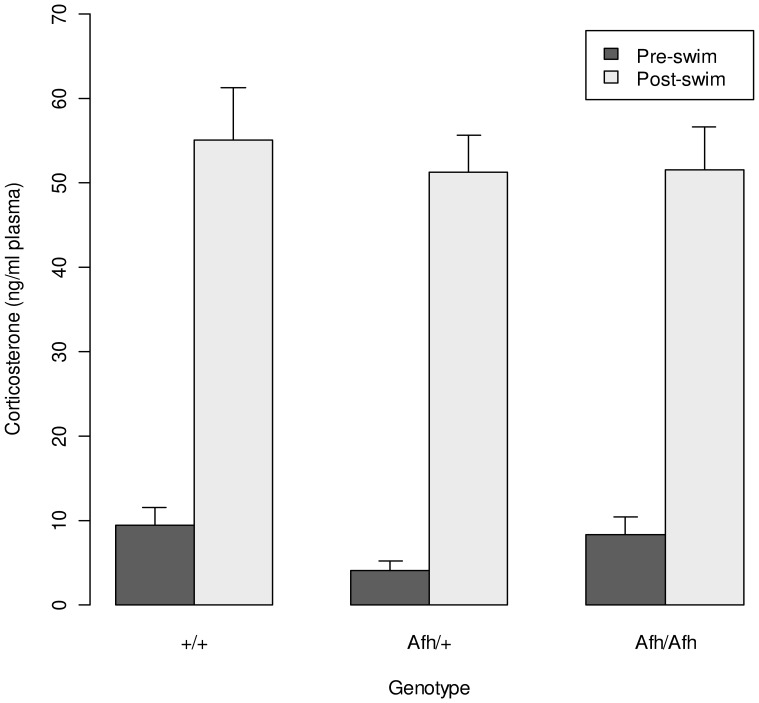
The corticosterone response to the forced swim test. A significant increase in plasma corticosterone level was seen following the forced swim (F = 160, P<0.0001) but there was no effect of genotype on either corticosterone level at baseline or in response to stress. (Data shown is mean ± SEM).

Following the first forced swim test, there was a significant increase in plasma corticosterone level (F = 211, P<0.0001), indicative of a stress response to the task. However, there was no effect of genotype on either baseline or the post-swim induced increase in plasma corticosterone level (Figure 4).

### Validation in Human Data

**Figure 5:**
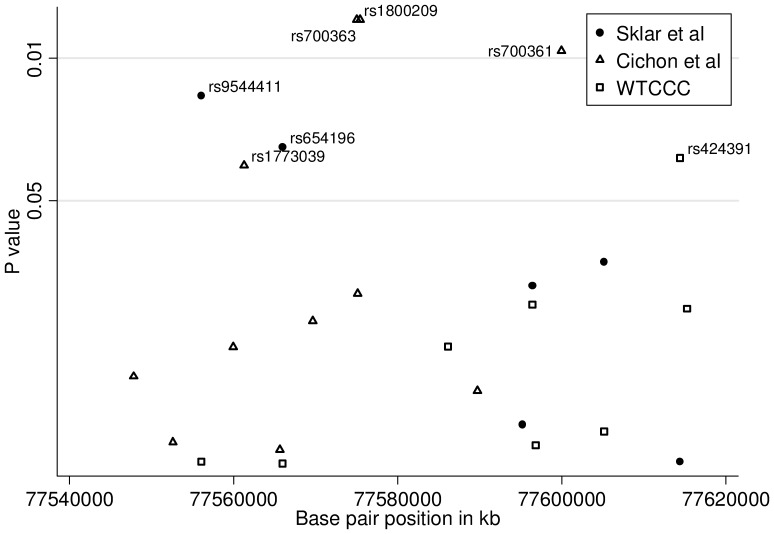
Evidence of association between SNPs in *FBXL3* and bipolar disorder in three independent samples (WTCCC [30], Sklar et al [31] and Cichon et al [32]).

We identified 20 SNPs genotyped in the WTCCC [30], Sklar et al [31] and Cichon et al [32] datasets occurring in *FBXL3* or 20 kb around the gene. Results for the 7 SNPs which reached a nominally significant p value (*P*<0.05) for association with bipolar disorder are given in Figure 5 and Table 1. The gene p values for *FBXL3* in the WTCCC [30] Sklar et al [31] and Cichon et al [32] datasets were 0.35, 0.02 and 0.04 respectively. This resulted in a significant result from a combined analysis of the three data sets (P = 0.009).

**Table 1:** Unadjusted SNP p-values for SNPs in FBXL3 reaching significance p<0.05 in at least one study.

			Cichon et al [32]	WTCCC [30]	Sklar et al [31]
SNP	Position	Allele	P value	P value	P value
rs700361	77599954	C/T	0.009	–	–
rs700363	77575396	G/A	0.006	–	–
rs1800209	77574983	A/G	0.006	–	–
rs424391	77614437	C/T	–	0.030	0.954
rs654196	77565972	A/G	–	0.979	0.027

We also tested SNPs in and 20 kb around a paralog of *FBXL3, FBXL3B* (also known as *FBXL21*). While in the WTCCC data one SNP (rs17701996) was significantly associated with bipolar disorder (*P*  = 7.1×10^−5^), SNPs in both Sklar et al [31] and Cichon et al [32] datasets were non-significant as were results from analyses of the resulting gene p values from the combined analysis.

## Discussion

This study is the first to characterise anxiety, depression-like and exploratory behaviour of *Afh* mice. Consistent with our hypothesis, and previous studies of circadian clock mutants, the behavioural characteristics of *Afh* mice (reduced anxiety and depression-like behaviour) were analogous to some features of human mania. Moreover, variation in the human orthologue of *Fbxl3* was associated with bipolar disorder in three clinical samples.

The open field, elevated plus maze and light-dark box were used to evaluate anxiety-like behaviour by measuring the spontaneous exploratory behaviour of *Afh* mice in aversive environments. *Afh*/*Afh* mice spent consistently more time in the anxiogenic areas of each paradigm and, in the open field test and light-dark box, entered these areas significantly more frequently when compared to their *Afh*/+ and +/+ littermates. There was no effect of genotype on exploratory behaviour in the holeboard test but there was some evidence for increased locomotor activity (measured as speed and distance travelled) in the light dark test and elevated plus maze.

The forced swim test was used to assess depression-like behaviour. While there was no effect of genotype on time spent immobile in the first trial, in the second trial *Afh/Afh* mice spent less than half the time immobile compared to *Afh/+* and +/+ mice. However, there was no effect of genotype on the stress response, as assessed by the rise in plasma corticosterone following the forced swim test. An increase in immobility over time is considered to reflect the development or learning of behavioural despair [40]. The failure of *Afh/Afh* mice to develop immobility indicates a specific reduction in depression-like behaviour rather than a simple change in the stress response in these mice.

The behavioural characteristics of *Afh* mice are consistent with those reported in other animal models with circadian clock disturbances. Tataroglu et al [23] reported that rats with lesions to the SCN failed to develop behavioural despair in the forced swim test. Similarly, mice lacking the circadian output molecule prokineticin2 (PK2) or over expressing *GSK3β* both result in decreased depression and anxiety-like behaviour [21], [24]. Similarities between our results and those reported for the ENU-induced clock mutant mouse (*Clock*) are the most striking. Roybal et al [26] reported that *Clock* mice show decreased anxiety-like behaviour in the open field test, elevated plus maze and light dark box and reduced depression-like behaviour in the forced swim test. In addition, consistent with our findings, Easton et al [22] reported that, while *Clock*/+ and +/+ developed increasing immobility over two trials of the forced swim test, the same effect was attenuated in *Clock/Clock* mice. *Clock* mutant mice have a very similar circadian phenotype to *Afh* mice. *Clock/Clock* and *Afh*/*Afh* mice have circadian periods of 27.36 and 26.7 hours respectively, while heterozygotes are more similar to wildtype mice (*Clock/*+, 24.2 hrs and *Afh*/+; 24.23 hrs). Consistent with effects of genotype on circadian dysfunction, the altered mood and anxiety -like behaviours in both *Afh* and *Clock* mutants appear to be limited to homozygotes. Future experiments to assess whether the behavioural phenotype observed in *Afh* mutants can be reversed by lithium or other mood stabilising drugs are clearly warranted for the validation of *Afh* as a model of mania.

The manic phase of bipolar disorder is characterised by disturbances in the circadian cycle and sleep and reduced depression and anxiety with hyperactivity. *Afh* mice have a previously well characterised circadian disturbance [28]–[29] and in the current study showed both reduced anxiety and depression-like behaviour. While significant hyperactivity was not observed in this study, we found some evidence to suggest that there was a trend for *Afh/Afh* mice to be more active in response to novelty, particularly in the light/dark box. *Afh/Afh* mice were also, on average, more than three grams heavier than their heterozygote and wildtype littermates. This association did not confound the effects of genotype on the behavioural measures and is consistent with previous reports of the *Clock* mutant. Turek et al [43] reported that *Clock/Clock* mice have increased appetite, weight gain and symptoms of metabolic syndrome. Similarly, obesity and metabolic disturbances have been consistently associated with bipolar disorder and may be the result of an overlapping genetic aetiology [44]–[45].

The lengthened circadian rhythm observed in *Afh* mice results from a Cys^358^Ser substitution in the protein encoded by *Fbxl3.* Using three genome-wide association data sets including more than 4,000 cases and 5,000 controls, we found that on a combined analysis, variation in *FBXL3,* the human orthologue of *Fbxl3* was associated with bipolar disorder. This association was particularly strong in the Cichon et al [32] dataset. These findings are novel and add to a growing body of literature linking variation in circadian clock genes with bipolar disorder [15]–[19]. However, we did not conduct a systematic meta-analysis and the results require replication across a larger, more exhaustive number of data sets.

The long circadian rhythm observed in both *Afh* and *Cloc*k mutant mice is analogous to the chronotype of ‘eveningness’ in humans [28], [46]. Interestingly, in line with our findings and those of circadian clock mutant mice, several recent reports have suggested that ‘eveningness’ (which is associated with variation in human circadian clock genes including *CLOCK* [47]) is a risk factor for bipolar disorder [2]–[4]. However, it remains unclear whether a central disruption of the circadian clock is directly involved in the pathogenesis of bipolar disorder, or circadian clock genes have pleiotropic effects on both circadian rhythms and mood [48].

A recent study reporting the effects of a region-specific knockdown of *Clock* provides tentative evidence to suggest that the effects of circadian clock genes on locomotor activity and on mood can be separated. Specifically, knockdown of *Clock* in the ventral tegmental area (VTA) was shown to result in an increase in dopaminergic neuron firing and a mixed state of mania and depression-like behaviour. However this behaviour was not accompanied by a long circadian rhythm. Rather, knockdown mice presented with a circadian period 15 minutes shorter than wildtype [49]. It is possible then that peripheral circadian clocks outside of the SCN in regions such as the VTA have effects on mood which are independent of circadian locomotor activity. Future studies should therefore consider region specific knockdown or knockout of *Fbxl3* in order to disentangle the gene’s effects on both mood and the central circadian clock.

Consistent with reports from other models of circadian dysfunction *Afh* mice exhibited a behavioural profile analogous to aspects of human mania. Moreover the gene responsible for this behaviour was associated with bipolar disorder in data taken from three large independent and well characterised association studies. The current study therefore provides further evidence to an expanding literature linking circadian rhythm dysfunction and bipolar disorder and identifies *FBXL3* as a potential candidate for study in human populations. The current study also suggests that, following further characterisation and validation, *Afh* may be a useful mouse model of some of the features of mania.
